# Domains of the cucumber mosaic virus 2b silencing suppressor protein affecting inhibition of salicylic acid-induced resistance and priming of salicylic acid accumulation during infection

**DOI:** 10.1099/vir.0.063461-0

**Published:** 2014-06

**Authors:** Tao Zhou, Alex M. Murphy, Mathew G. Lewsey, Jack H. Westwood, Heng-Mu Zhang, Inmaculada González, Tomás Canto, John P. Carr

**Affiliations:** 1 Department of Plant Sciences, University of Cambridge, Downing Street, Cambridge CB2 3EA, UK; 2 Department of Plant Pathology, China Agricultural University, 2 Yuanmingyuan West Rd, Beijing 100193, PR China; 3 State Key Laboratory Breeding Base for Zhejiang Sustainable Pest and Disease Control, Institute of Virology and Biotechnology, Zhejiang Academy of Agricultural Sciences, 198 Shiqiao Rd, Hangzhou 310021, Zhejiang, PR China; 4 Centro de Investigaciones Biológicas, CIB, CSIC, Ramiro de Maeztu 9, 28040 Madrid, Spain

## Abstract

The cucumber mosaic virus (CMV) 2b silencing suppressor protein allows the virus to overcome resistance to replication and local movement in inoculated leaves of plants treated with salicylic acid (SA), a resistance-inducing plant hormone. In *Arabidopsis thaliana* plants systemically infected with CMV, the 2b protein also primes the induction of SA biosynthesis during this compatible interaction. We found that CMV infection of susceptible tobacco (*Nicotiana tabacum*) also induced SA accumulation. Utilization of mutant 2b proteins expressed during infection of tobacco showed that the N- and C-terminal domains, which had previously been implicated in regulation of symptom induction, were both required for subversion of SA-induced resistance, while all mutants tested except those affecting the putative phosphorylation domain had lost the ability to prime SA accumulation and expression of the SA-induced marker gene *PR-1*.

Salicylic acid (SA) is required for elicitor-triggered immunity and establishment of systemic acquired resistance against a wide range of pathogens, including viruses (Palukaitis & Carr, 2008). SA induces several antiviral mechanisms, some of which may involve RNA silencing but none of which are well understood (Lee *et al.*, 2011; Lewsey & Carr, 2009). In *Arabidopsis thaliana* and tobacco (*Nicotiana tabacum*) resistance to systemic movement of cucumber mosaic virus (CMV) is induced by treatment with SA or its synthetic analogues (Mayers *et al.*, 2005; Naylor *et al.*, 1998; Smith-Becker *et al.*, 2003), or by inducing endogenous SA biosynthesis by pre-infection with an incompatible pathogen (Bergstrom *et al.*, 1982; Naylor *et al.*, 1998).

In *A. thaliana* and tobacco SA inhibits replication and cell-to-cell movement of some viruses, including tobamoviruses and potato virus X, but CMV evades this local resistance (Lee *et al.*, 2011; Mayers *et al.*, 2005; Murphy & Carr, 2002; Naylor *et al.*, 1998; Wong *et al.*, 2002). The ability of CMV to overcome SA-induced resistance to local movement and replication is conferred by the multifunctional 2b protein (Ji & Ding, 2001); the smallest (110 aa) of five proteins CMV is known to encode (Palukaitis & García-Arenal, 2003). Properties of the 2b protein include suppression of antiviral silencing (Brignetti *et al.*, 1998), which it impedes by binding double-stranded short-interfering RNAs (González *et al.*, 2010, 2012; Goto *et al.*, 2007; Harvey *et al.*, 2011; Wang *et al.*, 2011).

Increased SA biosynthesis has been associated with incompatible plant–pathogen interactions, especially those involving hypersensitive-type resistance and host cell death in the infection zone (Malamy *et al.*, 1990; Métraux *et al.*, 1990), or with abortive defence reactions involving systemic necrosis (Jovel *et al.*, 2011). However, SA accumulation can occur during some non-necrotizing, compatible plant–virus interactions (Love *et al.*, 2005; Whitham *et al.*, 2003), including infection by CMV of *A. thaliana* (ecotype Col-0). Expression of *ICS1* (a gene encoding a key SA biosynthetic enzyme; Wildermuth *et al.*, 2001), SA accumulation and expression of *pathogenesis-related (PR)-1* (a highly SA-responsive gene) (Cutt *et al.*, 1988) were all increased in leaves systemically infected with CMV (Lewsey *et al.*, 2010a). Experiments using a mutant lacking the *2b* gene (CMVΔ2b; Ryabov *et al.*, 2001) or plants constitutively expressing a *2b* transgene showed that the 2b protein is necessary but not sufficient for inducing SA accumulation. This suggested that the 2b protein facilitates or primes elicitation of SA biosynthesis by other viral gene product(s) (Lewsey *et al.*, 2010a). Contrastingly, the CMV P6 silencing suppressor inhibits SA-induced gene expression (Laird *et al.*, 2013).

We investigated various 2b protein domains for roles in evasion of SA-induced resistance or in priming of CMV-induced SA accumulation. We used CMV strain Fny (Roossinck & Palukaitis, 1990). This strain induces severe symptoms in tobacco and *A. thaliana* and expression of its 2b protein in transgenic plants generates strong developmental phenotypes (Lewsey *et al.*, 2009). Experiments were carried out using WT CMV, CMVΔ2b, eight CMV variants with a range of site-directed mutations in various 2b protein functional domains (González *et al.*, 2010; Lewsey *et al.*, 2009), and an additional site-directed mutant harbouring a deletion in the *2b* sequence encoding amino acids 62–65 (mutant ΔGSEL), corresponding to four conserved amino acids within the domain identified by Ye *et al.* (2009) as conditioning strong RNA silencing suppression by the 2b protein of the SD strain of CMV, although mutation of this sequence did not abolish silencing suppression by the Fny-CMV 2b protein. This is in a region thought to interact with the host silencing factor Argonaute 1 (González *et al.*, 2012). As noted previously, because of the overlap of the CMV 2a and 2b ORFs in CMV RNA 2, mutations in the *2b* gene affect the 2a protein sequence (Du *et al.*, 2008; Lewsey *et al.*, 2009). Although it is conceivable that this overlap could influence the effects of some of the CMV 2b mutants (with the exception of CMVΔ3T, which affects a sequence beyond the *2a/2b* gene overlap), we believe this is unlikely. This is because no evidence from previous studies using *2b*-transgenic plants or CMVΔ2b variants in which there are no deletions in the 2a reading frame (Ji & Ding, 2001; Lewsey *et al.*, 2010b) suggests any relationship(s) between the 2a protein, SA biosynthesis, or the subversion of SA-induced resistance.

Four-week-old CMV-susceptible tobacco plants (‘Xanthi’) were sprayed with 2 mM SA or with water [containing 0.11 % (v/v) ethanol] as the control treatment daily for 4 days prior to inoculation on lower leaves with virus. The 2b protein allows CMV accumulation to reach the same level in SA-treated as in control-treated inoculated leaf tissue (Ji & Ding, 2001; Lewsey & Carr, 2009). This was examined in greater detail by imaging infection sites on SA-treated and control leaves inoculated with previously described viral constructs expressing the GFP: CMV-GFP and CMVΔ2b-GFP (Soards *et al.*, 2002) at 4 days post-inoculation (Fig. 1a). As seen previously, cell-to-cell movement of CMV-GFP was unaffected by SA and, in the absence of SA, CMVΔ2b-GFP moved preferentially through mesophyll rather than through epidermal cells (Murphy & Carr, 2002; Soards *et al.*, 2002) (Fig. 1a). CMVΔ2b-GFP infection sites were difficult to locate on inoculated leaves of SA-treated plants and in those sites observed, GFP was localized to single epidermal cells (Fig. 1a). The behaviour of CMVΔ2b-GFP appeared similar to that of a GFP-expressing tobacco mosaic virus; cell-to-cell movement of which was inhibited in SA-treated tobacco (Murphy & Carr, 2002).

**Fig. 1.**
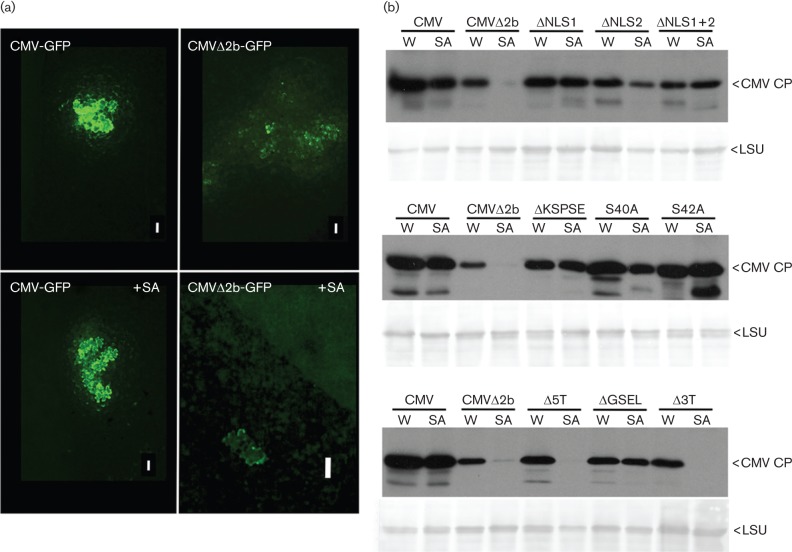
Effects of SA treatment on infection of tobacco leaves by CMV or CMV variants expressing mutant 2b proteins. (a) Epifluorescent microscopy images of infection sites of CMV expressing GFP (CMV-GFP) or CMVΔ2b-GFP on leaves of plants previously treated with SA (+SA) or untreated. Bars, 0.13 mm. (b) Immunoblot analysis for accumulation of WT CMV and a selection of 2b mutants. Results of three experiments are shown in which CMV coat protein (CP) accumulation in directly inoculated tobacco leaves, previously treated with SA or water (W) containing 0.11 % (v/v) ethanol, was compared for CMV and the indicated mutants. Equal protein loading verified by Ponceau S staining: the band for the large subunit (LSU) of ribulose 1,5-bisphosphate carboxylase is shown (lower panels).

Due to the difficulty of imaging CMVΔ2b-GFP in SA-treated tobacco plants, immunoblotting was used to detect virus accumulation in most experiments. A suspension of 5 µg ml^−1^ of purified virions of WT or mutant CMV was mechanically inoculated onto leaves as previously described (Naylor *et al.*, 1998: Soards *et al.*, 2002). WT and mutant CMV genomic RNAs were generated by *in vitro* transcription. Tobacco plants inoculated with these infectious RNAs were used to purify virions (Westwood *et al.*, 2013). Four days later, protein was extracted from inoculated leaves and analysed for virus accumulation, using CMV coat protein (CP) accumulation as a proxy, by SDS-PAGE and immunoblotting with anti-CMV CP (Lewsey *et al.*, 2009). Each mutant was examined at least three times for the effects of SA on its accumulation in inoculated leaves. In line with previous results (Ji & Ding, 2001; Lewsey & Carr, 2009) CMV accumulation in the inoculated leaves was unaffected by pre-treatment with SA but accumulation of CMVΔ2b was markedly inhibited (Fig. 1b). CMV variants carrying mutations in one or both elements of the bipartite, arginine-rich nuclear localization sequence (NLS) that overlaps the key RNA binding domain needed for silencing suppression (González *et al.*, 2012) (mutants ΔNLS1, ΔNLS2 and ΔNLS 1+2, respectively) were, like WT CMV, able to accumulate to readily detectable levels in SA-treated tissue (Fig. 1b). Similarly, mutant viruses possessing 2b proteins with alanine to serine substitutions in one of two putative phosphorylation sites (mutants S40A and S42A), or deletion of both sites (ΔKSPSE), as well as the ΔGSEL mutant were able to accumulate in SA-treated leaves (Fig. 1b). However, SA treatment did inhibit accumulation of the CMV mutants Δ5T, in which the RNA sequence encoding the N-terminal 17 aa of the 2b protein was deleted, and Δ3T, which expresses a 2b protein lacking its 16 C-terminal residues (Fig. 1b).

Paradoxically, although the 2b protein permits CMV to evade SA-induced resistance to local replication and movement (Ji & Ding, 2001; Lewsey & Carr, 2009; Naylor *et al.*, 1998), CMV infection increased expression of SA-regulated genes including *PR-1* in *A. thaliana* (Whitham *et al.*, 2003). These gene expression changes occurred as a result of increased SA accumulation, which is facilitated but not directly elicited by the 2b protein (Lewsey *et al.*, 2010a). We used immunoblotting (with an anti-PR1 serum cross-reacting with PRs 1a, 1b and 1c; Carr *et al.*, 1985) to examine accumulation of WT or mutant variants of CMV. Consistent with previous results in *A. thaliana* (Lewsey *et al.*, 2010a) we found that at 4 days post-inoculation PR1 accumulation was elevated in CMV-inoculated but not in CMVΔ2b-inoculated leaves of tobacco plants (Fig. 2a). Of the mutants tested, only CMVS40A, CMVS42A and CMVΔKSPSE retained the ability to elicit PR1 accumulation to levels comparable to, or slightly higher than, WT CMV (Fig. 2a).

**Fig. 2.**
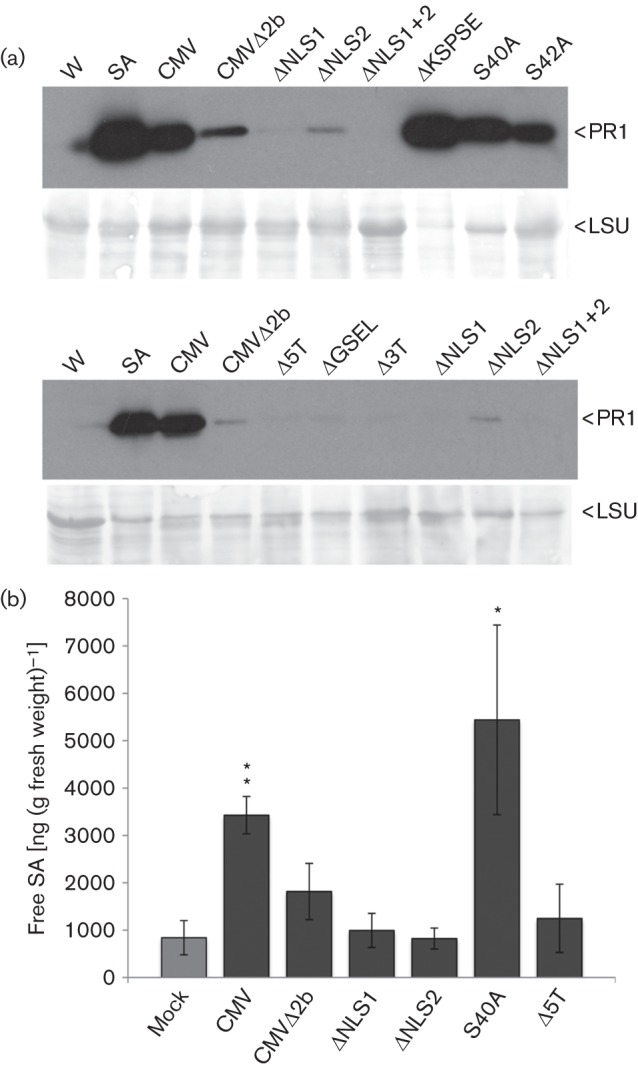
Effects of WT and mutant CMV on PR1 accumulation in tobacco plants. (a) Immunoblot analysis (using anti-PR1) of samples from two independent experiments comparing PR1 accumulation in leaves inoculated with CMV variants carrying mutant *2b* genes (indicated above each lane) or WT CMV, or mock-inoculated leaves treated with SA or water (W) containing 0.11 % (v/v) ethanol. Equal protein loading was verified by Ponceau S staining (lower panels). (b) SA accumulation measured by high-performance liquid chromatography in mock-inoculated or CMV- or mutant-inoculated leaves at 4 days post-inoculation. Significantly elevated SA levels (compared to basal SA accumulation in mock-inoculated leaves) indicated by *(*P*≤0.05) or **(*P*≤0.01) (Student’s *t*-test; *n* = three replicates).

PR1 accumulation is considered a reliable indicator for increased SA levels in tobacco (Malamy *et al.*, 1990). To check that this assumption is correct for CMV-infected tobacco, leaf extracts were analysed using high performance liquid chromatography (Surplus *et al.*, 1998). Analysis using a selection of CMV mutants indicated that PR1 accumulation correlated broadly with SA level (Fig. 2b). The highest SA levels were seen in leaves inoculated with the mutant CMVS40A (Fig. 2b). Interestingly, some PR1 accumulated in leaves infected with CMVΔNLS2 (Fig. 2a) but these leaves showed no apparent increase in SA (Fig. 2b), suggesting the existence of a weak, SA-independent *PR-1*-inducing mechan.

The mechanism by which the 2b protein facilitates SA accumulation is different from that by which the 2b protein of the HL strain induces increased reactive oxygen species production and necrosis in *A. thaliana*, since this function was not abolished by the deletion of the C-terminal 12–33 aa (Inaba *et al.*, 2011). Based on previous analysis of 2b protein domain function (González *et al.*, 2010), it appears that the ability of the 2b protein to prime SA and PR1 accumulation is unrelated to silencing suppression. Both NLS domains and the KSPSE domain are required for silencing suppression although mutations of individual phosphorylation sites in the KSPSE domain (S40A and S42A) do not abolish RNA silencing (González *et al.*, 2012). SA and PR1 levels increased in leaves inoculated with CMV variants carrying the S40A and the S42A mutations, as well as the KSPSE mutation. Using GFP-2b protein fusions, González and colleagues (2010) showed that WT 2b and the S40A and S42A mutants accumulate in nuclei and nucleoli to similar extents, whereas ΔKSPSE mutant 2b protein localizes predominantly to the nucleus and nucleolus and is absent from the cytoplasm. Overall, the data suggest that several 2b protein domains are required for priming of SA biosynthesis. Results obtained with mutations in the putative phosphorylation domain suggest that priming of SA accumulation by the 2b protein requires its accumulation within the nuclear or nucleolar compartments and that the presence of a functional phosphorylation domain limits the extent to which the 2b protein primes SA biosynthesis (Fig. 3).

**Fig. 3.**
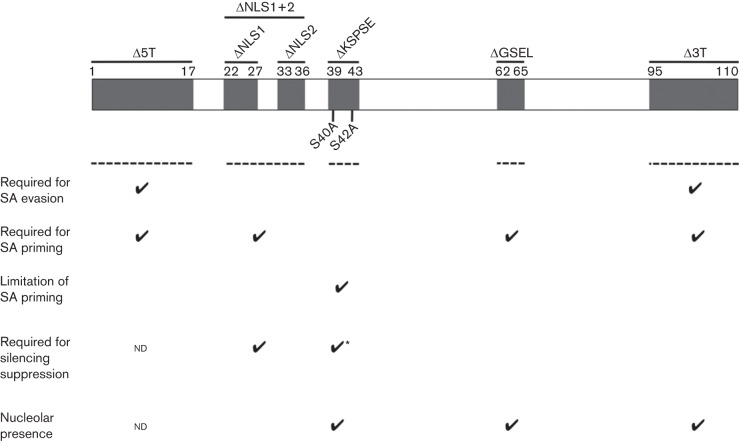
Roles of CMV 2b protein domains in evasion of SA-induced resistance or in priming of SA biosynthesis. Fny-CMV 2b protein map depicting known or putative functional domains investigated in this study (grey boxes) with mutation names and positions of mutations. Dotted lines indicate domains required for evasion of SA-induced resistance to replication and local movement (the N- and C-terminal domains), priming of CMV-induced SA accumulation (the N- and C-terminal domains, plus the NLS and GSEL), and limiting CMV-induced SA accumulation (putative phosphorylation domain: KSPSE). Information on roles of domains in RNA silencing suppression or nucleolar localization is from González *et al.* (2012). For the KSPSE domain, an asterisk indicates that deletion of the entire domain (not mutation of individual putative phosphorylation sites) abolishes silencing suppression (González *et al.*, 2012). ND, Not determined.

With respect to the roles of the N- and C-terminal domains in evasion of SA-induced resistance (Fig. 3), previous analyses showed that these domains, respectively, positively and negatively regulate symptom induction by CMV (Lewsey *et al.*, 2009). Thus, deletion of the RNA sequence encoding the C-terminal domain of the 2b protein resulted in the mutant CMVΔ3T, which induced more severe symptoms than WT CMV in three host species (tobacco, *Nicotiana benthamiana* and *A. thaliana*), whilst deletion of the N-terminal domain (CMVΔ5T) ameliorated symptoms (Lewsey *et al.*, 2009, 2010b). Mutation of 2b protein domains identified as affecting the binding of short-interfering RNAs and suppression of antiviral RNA silencing (mutants ΔNLS1, ΔNLS2, ΔNLS1+2 and ΔKSPSE; González *et al.*, 2010, 2012) did not compromise the ability of the virus to evade SA-induced resistance. This finding is consistent with previous data suggesting that RNA silencing does not play an indispensable role in SA-induced resistance to CMV (Lewsey & Carr, 2009). The mode(s) of action through which the C- and N-terminal regions of the 2b protein affect symptom expression remain unknown. The C-terminal domain is relatively unstructured although it appears to have Mg^2+^-binding properties (Gellért *et al.*, 2012). Other experiments *in vitro* and in yeast, respectively, indicated that the 2b protein N- and C-terminal domains have DNA binding properties and that the C terminus has transcriptional activation activity (Ham *et al.*, 1999; Sueda *et al.*, 2010). However, if the C-terminal domain of the 2b protein was exerting its effects on SA-induced resistance by interacting with host DNA sequences, then mutating the NLS domain of the protein would have compromised the ability of the ΔNLS1, ΔNLS2 and ΔNLS1+2 mutants to overcome SA-induced resistance. Since this did not occur, it suggests that the unknown cellular target(s) for the 2b protein (in its role as a suppressor of SA-induced resistance) lie outside of the nucleus. Interestingly, this contrasts with our analysis of 2b-primed SA synthesis, which suggests that for this role the 2b protein has a nuclear or nucleolar target.
